# New Insights into Potential Biomarkers in Patients with Mild Cognitive Impairment Occurring in the Prodromal Stage of Dementia with Lewy Bodies

**DOI:** 10.3390/brainsci13020242

**Published:** 2023-01-31

**Authors:** Niels Hansen, Caroline Bouter, Sebastian Johannes Müller, Christoph van Riesen, Eya Khadhraoui, Marielle Ernst, Christian Heiner Riedel, Jens Wiltfang, Claudia Lange

**Affiliations:** 1 Department of Psychiatry and Psychotherapy, University Medical Center Göttingen, 37075 Göttingen, Germany; 2 Department of Nuclear Medicine, University Medical Center Göttingen, 37075 Göttingen, Germany; 3 Institute of Diagnostic and Interventional Neuroradiology, University Medical Center Göttingen, 37075 Göttingen, Germany; 4 Department of Neurology, University Medical Center Göttingen, 37075 Göttingen, Germany; 5 German Center for Neurodegenerative Diseases (DZNE), 37075 Göttingen, Germany; 6 Neurosciences and Signaling Group, Institute of Biomedicine (iBiMED), Department of Medical Sciences, University of Aveiro, 3810-193 Aveiro, Portugal

**Keywords:** prodromal dementia with Lewy bodies, neuroimaging, biomarker, psychiatry

## Abstract

Background: Prodromal dementia with Lewy bodies (DLB) can emerge with the onset of mild cognitive impairment (MCI). Standard biomarkers can help identify such patients to improve therapy and treatment strategies. Our review aims to describe the latest evidence on promising biomarkers in prodromal DLB with MCI onset (MCI-LB). Methods: We selected articles on different biomarkers in MCI-LB from PubMed and conducted a narrative review. Results: We identified potentially promising clinical biomarkers, e.g., (1) assessing autonomic symptoms specifically, (2) describing the cognitive profile in several subdomains including executive and visual functions, and (3) measuring the speed of speech. In addition, we describe the measurement of seeding amplification assays of alpha-synuclein in cerebrospinal fluid as a relevant biomarker for MCI-LB. Electroencephalographic markers, as in calculating the theta/beta ratio or intermittent delta activity, or analyzing peak frequency in electroencephalography—methods also potentially useful once they have been validated in large patient cohorts. The 18F fluorodesoxyglucose positron emission tomography (FDG-PET) technique is also discussed to investigate metabolic signatures, as well as a specific magnetic resonance imaging (MRI) technique such as for the volumetric region of interest analysis. Conclusions: These biomarker results suggest that MCI-LB is a promising field for the use of biomarkers other than established ones to diagnose early prodromal DLB. Further large-scale studies are needed to better evaluate and subsequently use these promising biomarkers in prodromal DLB.

## 1. Prodromal Dementia with Lewy Bodies with MCI

Dementia with Lewy bodies is an important neurodegenerative disease with a unique cognitive profile encompassing fluctuations, deficits in visuoconstruction, and impaired attentional-executive functions being the second most frequent form of neurodegenerative dementia [1,2]. Differential diagnosis remains a challenge because there is considerable overlap in the clinical features of neurodegenerative diseases.

Three types of prodromal dementia with Lewy bodies have been recently investigated and research criteria established [3]. Prodromal DLB can present as mild cognitive impairment (MCI) (MCI-LB)-, delirium-, or psychiatric-onset. MCI and psychiatric symptoms are common subtypes in prodromal DLB, whereas the occurrence of delirium is much less common [4,5]. Delirium can also be difficult to distinguish from DLB itself, as a recent review showed [6]. Delirium episodes may precede the development of a full DLB pattern by months to years [7]. According to the research criteria of Mc Keith [3], MCI-LB consists of cognitive impairment in one or more cognitive domains. In addition, such cognitive impairment should be mainly due to disorders of attentional executive or visual processing. Prodromal DLB is characterized by the presence of the same core features as DLB, such as fluctuations in cognitive function, which include various pathophysiological mechanisms [8], recurrent visual hallucinations, rapid eye movement behavioral disorder (RBD), or symptoms of parkinsonism. Established biomarkers include (1) single photon emission computed tomography (SPECT) examination showing decreased dopaminergic uptake in nigrostriatal pathways, (2) polysomnographic confirmation of REM sleep without atonia, and (3) decreased uptake of meta-iodobenzylguanidine (MIBG) on cardiac scintigraphy. Probable MCI-LB can be diagnosed if two core features, or one core feature and an established biomarker are present. In contrast, only a possible MCI-LB can be diagnosed if only one core feature or two biomarkers are present. This enables us to classify an MCI-LB. However, it is often difficult to distinguish disease entities such as MCI-LB and MCI due to Parkinson’s disease, as both share a similar neuropsychological profile in which verbal and visuospatial memory retrieval is impaired, and memory encoding and storage are barely affected [9]. Therefore, biomarkers are needed to better distinguish such prodromal stages of different alpha-synucleinopathies.

As an established proposed biomarker, SPECT is suitable to identify only MCI-LB patients, albeit with only moderate sensitivity but high specificity compared to patients with MCI-AD [10]. The key criterion in this biomarker is decreased dopamine transporter uptake in the basal ganglia. The other very proven appropriate biomarker is polysomnographic elicitation and confirmation of REM sleep behavior disorder [3]. However, the combination of MCI and isolated REM sleep behavior disorder confirmed with polysomnography is a poorly reliable predictor of conversion from LB-MCI to manifest DLB within two years [11]. Moreover, caution is warranted with this biomarker, as 3 of 44 cases with prodromal REM sleep behavior disorder actually developed DLB [12]. MIBG scintigraphy is another important component as an indicative biomarker for prodromal DLB, which can be considered especially when SPECT does not reveal reduced uptake of dopamine transporters in the basal ganglia. A recent cross-sectional autopsy study demonstrated that the strength of residual cardiac innervation in autoptic studies correlated with cardiac MIBG uptake, and values of calculated early and delayed cardiac to mediastinum ratios [13]. Thus, overall, SPECT, MIBG scintigraphy, and polysomnography are among the proposed biomarkers for DLB in the prodromal stage, including MCI-LB. 

Several additional biomarkers are currently being discussed to improve classification, such as performing quantitative EEG, revealing slowing and frequency variability, or insular thinning or gray matter volume loss on MRI. Our review article is dedicated to describing recent developments in MCI-LB to promote early diagnosis and symptom management skills according to current treatment guidelines as summarized by Taylor et al. [2].

## 2. Methods

Our report is a narrative review. Our search methods included screening PubMed for articles employing the following terms alone or in combination: (1) biomarker, (2) dementia with Lewy bodies (or Lewy body dementia) or DLB and MCI or MCI-LB, (3) prodromal dementia with Lewy bodies and MCI, (4) dementia with Lewy bodies and mild cognitive impairment, and (5) prodromal dementia with Lewy bodies and mild cognitive impairment. We selected articles that addressed our research question involving novel biomarkers for the differential diagnosis of MCI-LB that were published between 2010 and 2022, with a focus on the years 2020–2022. In our narrative review, we aim to describe the main ideas for the potential new biomarkers. In the future, as the database grows, a systematic meta-analysis would be useful to evaluate whether a new potential biomarker is useful for the diagnosis and monitoring of MCI-LB.

## 3. Clinical Assessment Strategies

New strategies for assessing the symptoms that characterize prodromal DLB are currently being explored.

### 3.1. Autonomic Symptom-Assessment Strategies

A recent study [14] demonstrated that autonomic symptoms, in particular, are of potential interest in patients with MCI suspected of having prodromal DLB. Patients with MCI-LB exhibit more autonomic symptoms, such as higher orthostatic intolerance states and more secretomotor symptoms than patients with MCI caused by AD and normal aging [14]. In addition, MCI-AD patients did not reveal more autonomic symptoms than a control group [14]. Their study employed a composite score measuring autonomic symptoms with 31 items, which showed 92% sensitivity and 42% specificity in discriminating MCI-AD patients [14]. In another study, Hamilton et al. [15] showed that probable MCI-LB respond abnormally more often to the Valsalva maneuver; however, low orthostatic blood pressure is not a clear factor by which to differentiate AD from MCI-LB. The measurement of autonomic symptoms is therefore very relevant for the early diagnosis of MCI-LB. However, measuring autonomic symptoms, e.g., with the Autonomous Symptom Checklist (ASC) [16], remains a challenge. Furthermore, 80% of patients with MCI-LB present autonomic symptoms more frequently than those with MCI-AD [17]. In addition, gastrointestinal symptoms occurred in 73% of patients with MCI-LB, versus 36% in MCI-AD [17]. Other autonomic symptoms such as salivation, constipation, incontinence, diurnal or nocturnal hyperhidrosis were not more frequent in MCI-LB than MCI-AD, but they were more severe and prolonged [17]. It is also important to note that autonomic symptoms even precede memory loss in patients with DLB. These autonomic symptoms often include daytime hyperhidrosis and constipation [18]. It is therefore tempting to postulate that autonomic symptoms may even precede cognitive dysfunction such as MCI. In conclusion, evaluating autonomic symptoms is a promising clinical assessment strategy for early-stage MCI-LB when considering various symptoms and manifestations of autonomic symptoms such as orthostatic intolerance or secretomotor function.

### 3.2. Neuropsychological Assessment Strategies

A review of several studies also showed that when caused by prodromal DLB, MCI’s neuropsychological profile is mainly characterized by deficits in executive, visuospatial, and attentional functions [19]. Another recent investigation confirmed that patients with MCI-LB, in particular, performed poorly in visuoconstructive and executive functions [17]. In MCI-LB, not only was executive dysfunction observed, but so was a slowed processing speed. Surprisingly, visuospatial dysfunction in that study was not as dysfunctional in MCI-LB. In contrast, MCI patients who had AD exhibited a distinct amnestic profile [20]. Overall, neuropsychological testing in MCI-LB is a promising clinical strategy, confirming a similar neuropsychological profile as manifesting DLB, entailing deficits in executive, visuospatial, and attentional functions.

### 3.3. Speech Assessment Strategies

A further clinical characteristic of MCI-LB is the reduction of speech and the reduction in smoothness of speech with which prodromal DLB patients speak compared to patients with AD-associated MCI [21]. Machine learning models were used to show good discrimination between DLB and healthy controls (AUC: 0.88), and between DLB and AD patients (AUC: 0.77) based on language features [21]. These findings encourage detailed language evaluation when patients with suspected MCI-LB present in the clinical setting.

### 3.4. Olfactory Function Assessment

Another interesting clinical observation is that patients with MCI-LB suffered worse olfaction than AD-triggered MCI patients in a study with 38 probable MCI prodromal DLB, 19 possible MCI-LB, and 19 AD patients [22], supporting olfaction as a clinical feature to potentially help differentially diagnose DLB in its early stage from AD. Thus, we would recommend testing olfactory function in patients in which no further clinical hints for prodromal DLB are present, and in whom olfactory function is reported to be dysfunctional.

### 3.5. Inference of Clinical Assessment Strategies

There are thus several different clinical assessment strategies for patients with MCI-LB. One in particular should be highlighted in this context, namely that the strongest predictor for conversion to DLB in MCI patients was non-amnestic MCI versus amnestic MCI, and multidomain as opposed to single domain MCI [23]. The presentation of these studies illustrates that there are a number of clinical features that may become relevant for the differential diagnosis of prodromal DLB if the results can be confirmed in larger cohorts.

## 4. Biofluid Biomarker

Recently, a Quaking Induced Conversion assay for alpha-synuclein (RT-QuIC) was tested as a promising tool in cerebrospinal fluid (CSF) to assess alpha-synuclein in prodromal DLB. The RT-QuIC for alpha-synuclein amplification is a promising tool proven to identify patients with MCI at DLB’s prodromal stage compared to unimpaired controls, with 95% sensitivity of and 96% specificity. Its accuracy is 96% for discriminating 81 MCI-LB [24]. RT-QuiC is a method similar to PCR for misfolded proteins in that it utilizes seed-triggered assembly and conversion of a misfolded protein. Initially, this method was used for PrP scrapie, and subsequently for other misfolded proteins such as alpha-synuclein. The advantage is that very small amounts of a misfolded protein can be amplified and thus made detectable that otherwise would not have been measurable [25,26]. It is understood that small amounts of misfolded protein act as seeds, which in turn attract substrate molecules such as human monomeric α-synuclein. A growing synuclein aggregate is induced in this way, which is accompanied by a conformational change in the substrate in a seed-competent state. By applying the RT-QuIC method, misfolded alpha-synuclein can be made detectable in blood and CSF samples.

A recent meta-analysis showed 0.91 diagnostic sensitivity and 0.96 specificity of seeding amplification assays investigating CSF alpha-synuclein in Lewy body disease [27], thus supporting seeding amplification assays for alpha synuclein as promising biomarkers in DLB, and probably DLB’s prodromal stages. In this context, it is also very beneficial to detect α-synuclein of central origin by isolating brain-derived exosomes in peripheral blood, as the alpha-synuclein content in neuronal exosomes differs relevantly in various neurodegenerative diseases as studies [28,29] have shown.

Consistent with these investigations and observations, detecting α-synuclein from neuronal exosomes from blood or CSF is the first step, while the next involves augmenting the alpha-synuclein content via RT-QuiC, which seems to vary in different neurodegenerative diseases, i.e., as in DLB at the prodromal stage and in MCI-LB in particular. RT-QuIC is useful for distinguishing MCI-LB from cognitively unimpaired individuals [24], but no study has yet demonstrated its usefulness for distinguishing MCI-LB from other early neurodegenerative disorders. However, a recent neuropathology study [30] showed that RT-QuiC for alpha-synuclein identified DLB patients with cortex involvement with high sensitivity (97%), but not those with primary brainstem or amygdala involvement (sensitivity only 50%). Furthermore, Lewy body pathology [patients with PD, PD with AD, and DLB] has been distinguished from patients without Lewy body pathology with high specificity (94%) and sensitivity (100%) [30].

## 5. Electroencephalography

Recent studies have shown that electroencephalography (EEG) recordings in MCI-LB potentially offer new biomarking opportunities. Calculating the theta/alpha rhythm ratio proved to be associated with impairments in language, memory, and visuospatial abilities. In contrast, the theta/beta rhythm ratio showed an association with memory and executive functions. The results of Baik’s study [31] suggest that an elevated theta/beta rhythm ratio is a biomarker for DLB rather than AD. Mixed pathology is more likely to be reflected by an elevated theta/alpha ratio [31]. Other EEG features might be also relevant for biomarking MCI-LB with more prominent frontal intermittent delta activity in 22% compared to none in AD. MCI-DLB patients also presented a lower peak frequency and slower wave activity than AD patients [32]. In another study, augmented pre-alpha power and reduced beta power as well as slower frequency were shown to be a characteristic feature of MCI-LB vs. MCI due to AD [33]. EEG thus seems to yield several parameters that could serve to discriminate DLB from AD patients even at the MCI stage. In summary, several EEG biomarkers (peak frequency, slow wave activity, intermittent frontal delta activity) help to differentiate MCI-LB from other neurodegenerative diseases such as MCI due to AD. However, to date, no study has addressed EEG’s superiority as a biomarker for differentiating MCI-LB from other α-synucleinopathies.

## 6. Neuroimaging Biomarkers

### 6.1. 18F Fluorodesoxyglucose Positron Emission Tomography

A recent FDG-PET study [34] identified metabolic signatures that better distinguish MCI-LB patients from MCI patients with AD. MCI-DLB patients exhibited hypometabolism in the brain regions of the parieto-occipital cortex extending into the temporal lobes and thalamus to the substantia nigra. In MCI-LB, the medial and posterior cingulate metabolism was preserved compared to AD-based MCI. Moreover, MCI-LB patients exhibited increased hypometabolism in the substantia nigra compared to AD-associated MCI patients [34]. Higher medial temporal metabolism and low substantia nigra metabolism have an additive value in distinguishing prodromal DLB from AD [34]. There is also evidence that a technique combining the volumetric region of interest analysis semi-quantitatively, via distinguishing two main hypometabolic clusters, increased accuracy (90%) in diagnosing MCI-LB [35]. A higher medial temporalis to substantia nigra ratio is both highly sensitive (94%) and specific (83%) for discriminating MCI-LB from MCI-AD [34], and thus appears superior to other parameters such as the cingulate insular sign, which have only high specificity (90%) but no sensitivity [34].

### 6.2. Magnetic Resonance Imaging

MRI is another useful tool for diagnosing MCI-LB early [36]. In addition, segmental evaluation of MRI helps to differentiate the subgroups of prodromal DLB from each other: for example, the variant with a psychiatric symptom onset of prodromal DLB showed significant atrophy of the substantia innominata, which was not found in MCI-LB [4]. Kantarci’s working group showed that an atrophic Meynert’s nucleus basalis is a prominent feature of prodromal dementia with Lewy bodies [36], and there is longitudinal evidence that progressive atrophy was particularly obvious in cholinergically innervated regions, together with clinical progression to probable DLB [36]. These studies indicate that magnetic resonance imaging is more accurate in distinguishing different types of dementia with Lewy bodies in the prodromal stage and, therefore, is potentially a subtle tool for distinguishing between various forms of α-synucleinopathies. More research is needed to support this assumption.

## 7. Discussion

**Figure 1:**
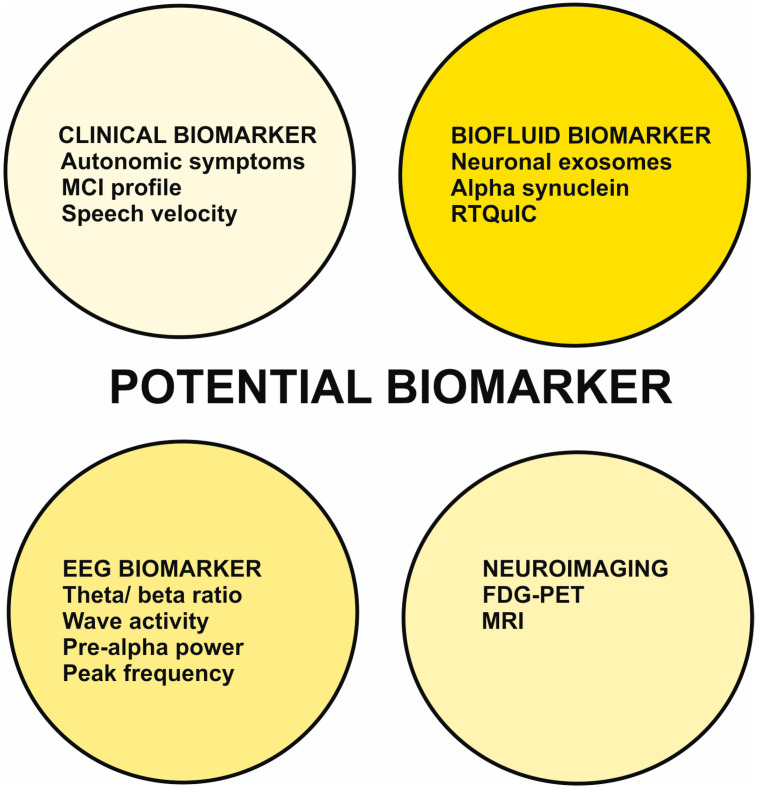
Potential biomarker of prodromal dementia with Lewy bodies with an MCI-onset.

In this report, we have illustrated recent developments in clinical features such as the presence of autonomic symptoms and speech velocity, and the cognitive-dysfunction profile, biomarkers such as seeding amplification tests of alpha-synuclein in CSF, EEG markers such as calculating the theta/ beta ratio or intermittent delta activity, as well as analyzing the peak frequency in EEG (Figure 1).

Abbreviation: FDG-PET = fluorodesoxyglucose positron imaging, MCI = mild cognitive impairment, MRI = magnetic resonance imaging, RTQuIC = Quaking Induced Conversion assay for alpha-synuclein.

FDG-PET and MRI imaging techniques have also been described (Figure 1), as have metabolic signatures and specific MRI techniques such as volumetric region of interest analysis for MCI-LB. To critically evaluate these new biomarkers in DLB in the prodromal stage with MCI, we briefly discuss them here and illustrate their advantages and disadvantages in Table 1.

Let us begin with the clinical biomarkers, which are generally promising tools [14,15] for both research and clinical practice because the assessment strategy is easy to apply, i.e., measuring autonomic symptoms via orthostatic tolerance or secretomotor symptoms, and using a score to assess autonomic symptoms. The disadvantage is the method’s lack of specificity, because, for example, orthostatic intolerance and secretomotor symptoms may coexist in patients with AD or other diseases. Other methods for assessing autonomic symptoms include the Valsalva maneuver, which is also easy to use but has several disadvantages in terms of its specificity, and that it may also reveal abnormalities in AD. Neuropsychological assessment is the crucial diagnostic step and does not seem to be a new biomarker. More importantly, it has also confirmed key features of the neuropsychological profile such as deficits in the executive, visuospatial, and attentional functions often already present in MCI-LB. More interesting in this context seems to be the study showing that multidomain MCI rather than the presence of amnestic or non-amnestic MCI could be relevant for the conversion of prodromal-stage DLB into DLB, which is of practical importance. Another potential tool of clinical nature and relevance is assessing language, which has yielded promising results. However, there is a critical overlap here with other neurodegenerative diseases revealing language impairments that are unlikely to be clearly distinguishable, i.e., primary progressive aphasias from the spectrum of frontotemporal lobar degeneration. Early loss of olfaction appears to be a general marker for specific neurodegenerative diseases that is not unique to MCI-LB but is likely to be detected in other alpha-synucleinopathies, but should be considered when the differential diagnosis is specific to AD. One promising biomarker is the examination of cerebrospinal fluid using RT-QuIC to determine alpha-synuclein levels. This method is both highly sensitive and specific, and may have a role to play in prodromal DLB’s diagnostic program in the future, but the determination method is complex and can only be performed in specialized tertiary care centers. Further studies suggest that not only can baseline EEG rhythm be used for biomarking, but that specific evaluations should then be carried out to extract the theta/beta rhythm ratio [31] or alpha/beta power [32], both of which are helpful but problematic in clinical practice. Here, measuring peak frequency and peak wave activity in EEG [33] might be simpler tools to help diagnose MCI-LB. Neuroimaging markers involving metabolism are also interesting but require specific knowledge for subsequent evaluation, such as metabolic signatures (metabolism in the medial and posterior cingulate) [34] or cluster analyses to distinguish hypermetabolic clusters in prodromal DLB [35]. Therefore, more research should be conducted on strategies that are easy to apply and do not take much time. Although manual segmentation is beneficial for distinguishing subclusters of DLB in the prodromal stage [4], the strategy is still time-consuming and not really feasible as a clinical strategy. In MRI, observing atrophic primary cholinergic innervation structures is a potentially promising new diagnostic measure. However, large cohorts are needed to test the diagnostic efficacy and safety of these methods. In Table 2, we summarize the currently standard biomarkers and potential new biomarkers in prodromal-stage DLB, along with the expected outcomes and the suggested mechanisms underlying the biomarker.

We have not addressed other promising biomarkers such as alpha-synuclein radiotracers-like compounds (18F) BF227 and (18F) WC-58 [37,38] because they have not been adequately investigated in DLB patients at the prodromal stage. Another interesting aspect is using neuroimaging biomarkers to assess neuroinflammation in prodromal-stage DLB tau pathology and to see how neuroinflammation interacts with DLB [39]. However, the proportion of glial neuroinflammation at DLB’s prodromal stage remains unclear, making it immensely important to measure glial neuroinflammation in prodromal DLB. However, no study has applied (11C)-PK11195 positron PET imaging as a proxy for microglial neuroinflammation in the brain in MCI-LB, which should be investigated in future studies. The fronto–temporo–parietal cortexes are a brain region of great interest as well for further studies, as these regions appear to be affected in early DLB patients also [40].

### 7.1. Limitations

An important study limitation is that the group to which we compare MCI-LB patients is an MCI due to AD group. We could not address other neurodegenerative diseases such as MCI due to FTLD or MCI of non-progressive character in this study because of the lack of data. Further research is needed to better assess the validity of novel biomarkers, not just by comparing MCI due to AD. Another important point to mention is that we have not discussed any biomarkers useful for monitoring therapeutic interventions because the study cohorts are often small. However, many of the biomarkers we discuss could theoretically serve as biomarkers for evaluating therapeutic interventions, such as imaging markers, e.g., FDG-PET, biofluid markers involving RTQuIC measurement of alpha-synuclein, or rapidly interpretable clinical symptomatology such as autonomic symptoms. Another difficult aspect that our biomarker article does not address is the distinction between MCI-LB and delirium-LB, which is often challenging because delirium and DLB may share similar features (for a review, see [6]).

### 7.2. Conclusions

Several promising potential clinical and molecular biomarkers have been discussed, that would facilitate the diagnosis of MCI-LB. The current landscape of biomarkers is stimulating and encouraging for investigators planning large-scale studies to enable the early diagnosis of DLB at its prodromal stage, with strong implications for treatment options.

## Figures and Tables

**Table 1:** Critical appraisal of different novel biomarking strategies for prodromal DLB with MCI onset.

Biomarker	Advantage	Disadvantage	References
**Autonomic symptoms**			
Higher orthostatic intolerance	Early and easy tool High sensitivity	Not specific to DLB, could also occur in AD Moderate specificity	[14]
More secretomotor symptoms	Early and easy tool High sensitivity	Not specific to DLB, could also occur in AD Moderate specificity	[14]
Composite score	Early and easy tool High sensitivity	Not specific to DLB, could also occur in AD Moderate specificity	[14]
Valsalva maneuver	Easy tool	Unspecific, Valsalva maneuver can be abnormal in many other diseases	[15]
**Neuropsychological testing**			
Executive, visuospatial and attentional deficits	Confirm research criteria	No novel biomarker, but biomarker is not good assessed in its early stages of DLB Belongs to established diagnostic criteria	[17]
Amnestic vs. Non-amnestic profile	Extends knowledge that exists for DLB vs. AD	Not novel biomarker	[20]
Multidomain vs. single domain	Helpful for prognosis prediction	Not helpful for prognosis per se	[23]
**Speech assessment**			
Reduction of speech and reduction of smoothness of speech	Easy tool	Overlap with other forms of cognitive decline and neurodegeneration such as forms of FTLD such as PPA Unspecific	[21]
**Olfaction**			
Reduction in olfaction	Easy tool	Unspecific Other alpha synucleinopathies might be not differentiated only AD, but also in AD it might occur	[22]
**Biofluid measurement**			
CSF RT-QuIC of alpha synuclein	High sensitivity and specificity	Method is complex and highly sophisticated	[24,27,30]
**EEG**			
Theta/beta rhythym ratio	Easy tool if EEG available	Additional evaluation is needed	[31]
Peak frequency and wave activity	Easy tool if EEG available No additional evaluation strategy, no time consuming method	Unspecific	[32]
Alpha or beta power	Easy tool if EEG available	Additional evaluation is needed	[33]
**Neuroimaging**			
**FDG-PET**			
Metabolic signature (preserved medial and posterior cingulate metabolism)	Available tool in tertiary care centers	Investigation with rays	[34]
Hypermetabolic cluster differentiation	Available tool in tertiary care centers Accurate	Additional strategy for evaluation is required	[35]
**MRI**			
Manual segmentation strategy	Good discrimination of subgroups of prodromal DLB with their atrophy profile	Unspecific for all prodromal DLB subtypes, time consuming and not good for discrimination of MCI due to DLB vs. AD	[4]

Abbreviations: AD = Alzheimer’s disease, DLB = dementia with Lewy bodies, EEG = electroencephalography, FTLD =Frontotemporal lobar degeneration, PPA = Primary progressive aphasia.

**Table 2:** Synopsis of established and potential biomarker of prodromal dementia with Lewy bodies with MCI-onset.

Standard Biomarkers	Potential Findings/Mechanisms in DLB and MCI-LB
123I-FP-CIT SPECT	Reduced nigrostriatal dopaminergic uptake due to nigrostriatal degeneration
Polysomnography	REM sleep behavior disorder as core clinical DLB feature
[^123^I] MIBG cardiac scintigraphy	Cardiac MIBG uptake reduced—cardiac noradrenergic loss of innervation and/or function
EEG	Dominant frequency variability, slowing of occipital basic rhythm activity—anomalies in neural synchronization, marker of cholinergic system integrity
MRI	Intact temporal lobe structures, cortical insular thinning, gray matter volume loss in anterior cingulum and medial frontal structures
FDG-PET	Occipital hypometabolism, relative preservation of the posterior cingulate mechanism (cingulate island sign)
Potential biomarkers	
Autonomic symptom assessment	Autonomic dysfunction
Neuropsychological assessment	MCI, attentional-executive dysfunction, impaired visuoconstruction as clinical DLB features related to dysfunctional regional and interregional brain networks
Speech assessment	Speech abnormalities
Olfactory function	Olfactory dysfunction caused by neurodegeneration in olfactory pathways
RT-QiC	α- synucleinopathy
Neuronal exosomes	α-synucleinopathy
FDG-PET	Higher medial temporalis to substantia nigra ratio
MRI	Atrophic substantia inominata, Nucleus basalis Meynert due to neurodegeneration in these structures
EEG	Peak frequencies of brain waves, regional or general continuous or discontinuously decelerated brain waves cause by brain-function impairments

Table 1 was drafted by relying on the following references [3,4,5,6,7,24,27,30,31,32,33,34,35,36]. Abbreviations: 123I-FP-CIT SPECT = (123)-I-2-ß-carbomethoxy-3ß-(4-iodophenyl)-N-(3-fluoropropyl) nortropane single photon emission computed tomography, DLB = dementia with Lewy bodies, EEG = electroencephalography, FDG-PET = fluorodesoxyglucose-positron emission tomography, MCI = mild cognitive impairment, MCI-LB = prodromal dementia with Lewy bodies with an MCI-onset, MIBG scintigraphy = [^123^I] Metaiodobenzylguanidine (MIBG) cardiac scintigraphy, MRI = magnetic resonance imaging, RT-QuiC = Quaking Induced Conversion assay for alpha-synuclein.

## Data Availability

No data was generated for this review and is therefore not available.
